# A Simple Protocol for Periodic Live Cell Observation of Flagellate Stages in the Lichen Alga *Trebouxia*

**DOI:** 10.21769/BioProtoc.5566

**Published:** 2026-01-20

**Authors:** Enrico Boccato, Fabio Candotto Carniel, Mauro Tretiach

**Affiliations:** Department of Life Sciences, University of Trieste, Trieste, Italy

**Keywords:** Lichen photobionts, Light microscopy, Live-cell microscopy, Microalgal culture techniques, Motile cells, Reproductive stages, *Trebouxia*

## Abstract

Flagellate stages of green microalgae such as *Trebouxia* are only partially characterised, with recent evidence suggesting that they are involved in both sexual and asexual reproduction. Conventional methods based on fixed samples in light, confocal, or electron microscopy provide only static observations and prevent real-time monitoring of living cells. To overcome this limitation, we have developed a simple and cost-effective protocol for observing *Trebouxia* flagellate cells over several days by coating microscopy slides with Bold’s basal medium. The method preserves cell viability and allows repeated imaging of motile cells in the same areas so that their behaviour and development can be continuously observed. In this way, qualitative observations, such as flagellate cell release, motility, and gamete fusion, can be combined with quantitative analyses of cell morphology. The protocol has proven to be robust and reproducible and was applied to several *Trebouxia* species. Compared to existing techniques, it allows the monitoring of dynamic processes and provides a powerful tool to study specific life stages not only in *Trebouxia* but also in other unicellular and colonial green algae.

Key features

• This protocol allows real-time monitoring over several days of *Trebouxia* flagellate cells with standard light microscopy.

• This protocol preserves cell viability and motility for repeated daily observations of the same cell groups.

• This protocol is simple, low-cost, and adaptable to other motile algal cells.

• This protocol is based on the methodology described in [1], where it was originally applied and validated.

## Graphical overview


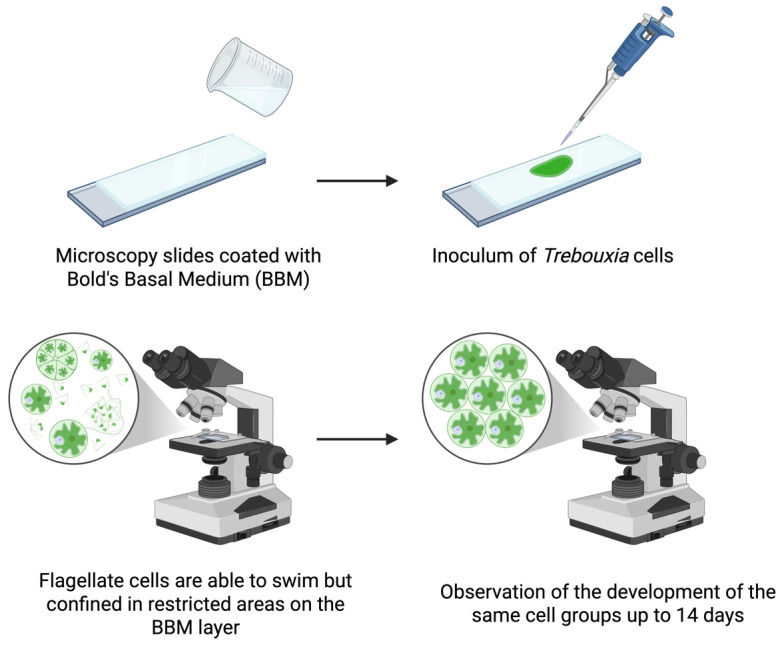


**Workflow for observation of flagellate cells of *Trebouxia* over several days**

## Background

*Trebouxia* Puymaly (Trebouxiophyceae, Chlorophyta) is a genus of unicellular, aero-terrestrial green microalgae whose representatives are the photosynthetic partner (i.e., photobiont) to about 50% of all lichen species [2]. The study of *Trebouxia* in culture is of fundamental importance as key traits for species identification, such as chloroplast morphology or specific stages of the life cycle, are difficult or impossible to observe in the lichenised form [3]. *Trebouxia* can reproduce asexually via autospores and aplanospores (non-motile) and via flagellate zoospores (motile). While the non-motile stages of the life cycle have been extensively studied for their taxonomic traits [4,5], the motile stages have been neglected over the years, so information on sexual reproduction in *Trebouxia* is limited. Sexual reproduction, which is common in other green algae such as Chlorophyceae and Ulvophyceae [6], is considered rare or absent in Trebouxiophyceae [7]. However, recent genome and transcriptome analyses have demonstrated the presence of meiotic genes in some representatives, suggesting possible sexual reproduction [6,8]. A recent study on *Trebouxia lynniae* [9], using flow cytometry and confocal techniques, showed that mature vegetative cells have twice the DNA content of flagellate cells, which were hence interpreted as gametes. This indicates the presence of sexual reproduction and challenges the current knowledge that flagellate cells only play a role in asexual reproduction. Further studies are thus clearly needed. A major limitation of previous studies was the common practice of fixing flagellate cells for light, confocal, or electron microscopy observations, which restricts the observed cells to a static state and prevents real-time observation of their behaviour or development. To overcome this limitation, a simple, cost-effective method has been developed to allow observation of living *Trebouxia* flagellate cells over several days [1]. With this method, the movement of the cells is spatially confined without compromising their viability, enabling the recording of images and videos. Compared to existing approaches, the new method allows detailed observation of cell development, motility, morphological differentiation, and interactions, including zoospore release and gamete fusion, providing a powerful tool to study asexual and sexual reproductive processes in *Trebouxia* and possibly other microalgae.

## Materials and reagents

**Biological materials**

1. *Trebouxia decolorans* Ahmadjian [Culture Collection of the University of Trieste (Italy), originally isolated from *Xanthoria parietina* (L.) Th. Fr.]

2. *T. gelatinosa* Ahmadjian [Culture Collection of the University of Trieste (Italy), originally isolated from *Flavoparmelia caperata* (L.) Hale]

3. *T. vagua* Voytsekhovich & Beck [Culture Collection of the University of Trieste (Italy), originally isolated from *Bagliettoa marmorea* (Scop.) Gueidan & Cl. Roux]

4. *T. angustilobata* (Beck) Beck (SAG, strain number: 2204)

*Note: All* Trebouxia *strains were cultivated on solid* Trebouxia *medium (TM, see Recipes) in Microbox Junior 40 vessels and subcultured every 3 weeks. Vessels were kept in a thermostatic chamber at 16 ± 1 °C and 23 ± 1 μmol photons·m^-2^·s^-1^ with a 12/12 h light/dark regime [1].*

**Reagents**

1. ZnSO_4_·7H_2_O (Carlo Erba, catalog number: 494907)

2. MnCl_2_·4H_2_O (Honeywell Fluka, catalog number: 63543)

3. MoO_3_ (Sigma-Aldrich, catalog number: 267856)

4. CuSO_4_·5H_2_O (Sigma-Aldrich, catalog number: 939315)

5. Co(NO_3_)_2_·6H_2_O (Supelco, catalog number: 1.02536)

6. EDTANa_2_ (Sigma-Aldrich, catalog number: 27285)

7. KOH (Sigma-Aldrich, catalog number: 484016)

8. FeSO_4_·7H_2_O (Honeywell Fluka, catalog number: 12354)

9. H_2_SO_4_, 96% (Carlo Erba, catalog number: 410261)

10. NaNO_3_ (Carlo Erba, catalog number: 481757)

11. MgSO_4_·7H_2_O (Carl ROTH, catalog number: 8283.1)

12. NaCl (Applichem, catalog number: APA29425000k)

13. K_2_HPO_4_ (VWR, catalog number: 26930.260)

14. KH_2_PO_4_ (VWR, catalog number: 26936.260)

15. CaCl_2_·2H_2_O (Supelco, catalog number: 1.02382)

16. H_3_BO_3_ (Sigma-Aldrich, catalog number: B6768)

17. Casein yeast peptone (Sigma-Aldrich, catalog number: 39396)

18. D-(+)-glucose (Sigma-Aldrich, catalog number: G7021)

19. Agar (Sigma-Aldrich, catalog number: 05040)

**Solutions**

1. Trace elements stock solution (see Recipes)

2. EDTA stock solution (see Recipes)

3. Fe stock solution (see Recipes)

4. Bold’s basal medium (BBM) (see Recipes)

5. *Trebouxia* medium (TM) (see Recipes)

**Recipes**

The BBM is prepared starting from the stock solutions listed in the Reagent column of Recipe 4. For the trace elements, EDTA, and Fe solutions, detailed recipes are provided below (see Recipes 1–3). All the following stock solutions can be stored at 4 °C in glass bottles for at least 2–3 months after preparation. We recommend using autoclaved tips each time an aliquot is taken from the stock solutions and using different tips for each solution to avoid possible contamination and cross-contamination. If contamination is detected in a stock solution, discard it and prepare a fresh one before using it for the preparation of BBM or TM.

**1. Trace elements stock solution**

**Table:** 

Reagent	Final concentration	Quantity or volume
ZnSO_4_·7H_2_O	8.82 g/L	0.441 g
MnCl_2_·4H_2_O	1.44 g/L	0.072 g
MoO_3_	0.71 g/L	0.0355 g
CuSO_4_·5H_2_O	1.57 g/L	0.0785 g
Co(NO_3_)_2_·6H_2_O	0.49 g/L	0.0245 g
dH_2_O		To a final volume of 50 mL

*Note: It may be necessary to autoclave the solution for all the reagents to dissolve.*

**2. EDTA stock solution**

**Table:** 

Reagent	Final concentration	Quantity or volume
EDTANa_2_	50 g/L	2.5 g
KOH	31 g/L	1.55 g
dH_2_O		To a final volume of 50 mL

**3. Fe stock solution**

**Table:** 

Reagent	Final concentration	Quantity or volume
FeSO_4_·7H_2_O	4.98 g/L	0.249 g
H_2_SO_4_ (96%)	1 mL/L	0.05 mL
dH_2_O		To a final volume of 50 mL

**Caution:** Always add acid to water and never water to acid!

**4. Bold’s basal medium (BBM) [10]**

**Table:** 

Reagent	Stock concentration	Volume (for 1 L)
NaNO_3_	25 g/L	10 mL
MgSO_4_·7H_2_O	7.5 g/L	10 mL
NaCl	2.5 g/L	10 mL
K_2_HPO_4_	7.5 g/L	10 mL
KH_2_PO_4_	17.5 g/L	10 mL
CaCl_2_·2H_2_O	2.5 g/L	10 mL
H_3_BO_3_	11.4 g/L	1 mL
Trace elements stock solution	-	1 mL
EDTA stock solution	-	1 mL
Fe stock solution	-	1 mL
dH_2_O		To final volume of 936 mL

Store in a glass bottle at 4 °C. It is better to have freshly prepared BBM every month, to be sure that all mineral nutrients are available to the algae when inoculation takes place and to minimise the possibility of contamination.

Prepare solid BBM according to Recipe 4 [10]. Transfer all solutions into a 1 L glass bottle and add 500 mL of dH_2_O. Place a magnetic stirring bar in the bottle and put it on a magnetic stirrer. Gradually add agar (15 g/L) while stirring and wait until it is evenly suspended from the bottom of the bottle before adding more. Note that it will only dissolve during autoclaving. Add dH_2_O to reach a final volume of 1 L.

**5. *Trebouxia* medium (TM)**

**Table:** 

Reagent	Final concentration	Quantity or volume (for 1 L)
NaNO_3_	0.5 g/L	0.5 g
Casein yeast peptone	10 g/L	10 g
D-(+)-glucose	20 g/L	20 g
Agar	15 g/L	15 g
BBM	-	To final volume of 1 L

Prepare TM according to Recipe 5 [11]. Pour 500 mL of liquid BBM into a 1 L glass bottle, add a magnetic stirring bar, and place it on a magnetic stirrer. Gradually add NaNO_3_ (0.5 g/L), casein yeast peptone (10 g/L), and D-(+)-glucose (20 g/L) to BBM while stirring until all reagents are completely dissolved. To prepare solid TM, gradually add agar (15 g/L) while stirring and wait until the agar is evenly suspended before adding more. Note that agar will only dissolve during autoclaving. Add BBM to reach a final volume of 1 L.

*Note: Due to the presence of organic nutrients, TM is prone to contamination. Always prepare it fresh before use. This recipe is prepared according to the original formulation described in [11], which is based on Bold’s basal medium (BBM). TM is used only for maintenance of* Trebouxia *strains prior to the experiments; all microscopic observations are performed in BBM (see Recipe 4).*

**Laboratory supplies**

1. 9-cm diameter Petri dishes (Sarstedt, catalog number: 82.1473.001)

2. 15 mL Falcon^®^ tubes (Corning, Falcon^®^, catalog number: 352096)

3. 100% cellulose adsorbent paper (Aurora, model: Soft 850)

4. Magnetic stirring bar

5. Microbox Junior 40 vessels (Sac O_2_, model: O95/40+OD95)

6. Microscopy slides (Menzel-Gläser, model: 76 × 26 mm, ground edges)

7. Plastic plug obtained by cutting the base of a syringe needle (Terumo, catalog number: AN*2332R1)

8. Razor blades (Gillette, model: silver platinum plus)

9. Sterile disposable loops (VWR, catalog number: 612-9358)

10. Sterile disposable syringes (Terumo, catalog number: MDSS20ESE)

11. Steripack^®^ pouches (PMS, catalog number: FP3050)

## Equipment

1. 0.5–5 mL volume pipette (Eppendorf, catalog number: 3123000071)

2. 0.5–10 μL volume pipette (Eppendorf, catalog number: 3123000020)

3. 200 and 600 mL beakers (VWR, model: Borosilicate 3.3 glass line)

4. Analytical balance (Sartorius, model: Research)

5. Autoclave (Fedegari, model: FVS 1)

6. Biological hood (BIOAIR, model: TopSafe 1.5)

7. Light microscope (Zeiss, model: Primostar 3) equipped with camera (Zeiss, model: Axiocam 208 colour) and 40× objective (400× magnification)

8. Magnetic stirrer (ALC Apparecchi per Laboratori Chimici Srl, model: mivar)

9. Nylon net of 40 μm mesh (Spectrum Labs, Spectra/Mesh^®^ Woven Filters)

10. Thermostatic chamber with adjustable temperature (15–20 °C) and dimmable, automated light control providing approximately 20 μmol photons·m^-2^·s^-1^ under a 12/12 h light/dark regime

11. Water distiller (Adrona, model: Crystal Ex)

## Software and datasets

1. Axiocam 208 colour native software (Zeiss, version 1.3.8)

2. Fiji software (ImageJ, version 1.54f)

3. R software (v. 4.3.2; R Core Team, 2023)

## Procedure

**A. Preparation of the slides for light microscopy observations**

1. Prepare Bold’s basal medium (BBM) following the instructions given in Recipe 4. When the agar is well suspended (5–10 min depending on the amount), remove the stirring bar and autoclave the bottle along with razor blades, a 200 mL beaker, a 600 mL beaker containing slides, 5 µL and 5 mL pipette tips, and pieces of nylon net of 40 μm mesh. Wrap razor blades, nylon nets, and beakers individually in aluminium foil. Place them inside Steripack^®^ pouches and seal them using a heat sealer before autoclaving. Use a standard sterilisation cycle (21 min at 121 °C and 1.1 bar).

2. Place 9-cm diameter Petri dishes, disposable syringes, and disposable loops in the biological hood and expose them to UV light for 20 min to sterilise the external surfaces of the packages and the working environment. Then, switch on the hood’s laminar flow for a further 20 min.

*Note: Only open sterile Petri dishes in the biological hood.*

3. When the sterilisation cycle of the autoclave is complete, transfer all sterilised materials to the biological hood.

**Caution:** Wait until the BBM has cooled down to at least 90 °C to avoid handling boiling liquids. Do not wait until the temperature drops below 60 °C; otherwise, the agar will begin to solidify.

**Figure 1.**
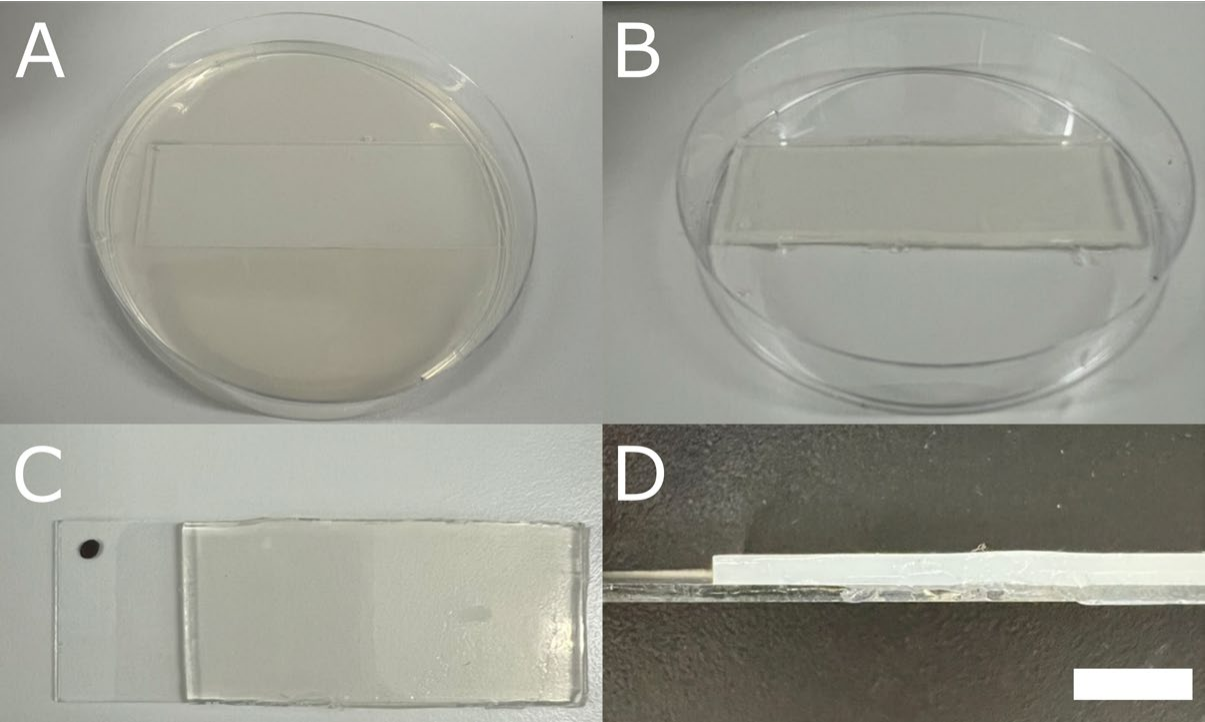
Preparation of Bold’s basal medium (BBM)-coated microscopy slides. (A) Petri dish with a microscopy slide in it, covered with solidified BBM. (B) The appearance of the BBM-coated microscopy slide after trimming the BBM in excess along the edges. (C) Slide marked with a waterproof marker to ensure a constant orientation during microscopy observations. (D) Lateral view of a BBM-coated slide, with a BBM layer of 2 mm. Scale bar = 5 mm.

4. Place a glass microscopy slide in a Petri dish and pour approximately 25 mL of hot, liquid BBM, so that the slide is covered with a 2 mm thick layer (Figure 1A, D).

*Note: A thinner layer will dehydrate too quickly, while a thicker layer will result in dark and indistinct images due to poor light transmission.*

5. Once the BBM has solidified, trim along the edges of the slide with a sterile razor blade and discard the excess medium (Figure 1B).

**Pause point:** The slides can be sealed in Petri dishes with Parafilm^®^ (while in the biological hood) and stored at 4 °C for up to 3–4 weeks.

6. Remove a small portion of the BBM layer from one side of each slide to expose the glass surface, then mark the slide (e.g., with a waterproof marker) to ensure constant orientation during microscopy observations (Figure 1C, D). This allows reliable use of the microscope’s coordinate system and tracking of the same cell groups over several days.

7. Moisten absorbent paper with sterile dH_2_O until it is evenly moist but not dripping.

8. Line the bottom of the Petri dishes containing the slides with the moist paper to ensure high humidity during the experiments.

*Note: Although this step ensures high humidity, it will cause the system to lose its sterility. However, BBM is a medium with only mineral nutrients, so it does not contaminate quickly; the system should be contamination-free for up to 14 days.*

**B. Preparation of TM and vessels for maintenance of *Trebouxia* strains**

1. Place the Microbox Junior 40 vessels inside Steripack^®^ pouches and seal them using a heat sealer.

2. Prepare TM following the instructions given in Recipe 5. When the agar is well suspended, remove the stirring bar and autoclave the bottle together with the vessels. Use a standard sterilisation cycle (21 min at 121 °C and 1.1 bar).

3. When the sterilisation cycle is complete, transfer all sterilised materials to the biological hood.

**Caution:** Apply the same safety precautions described for handling hot BBM when working with TM.

4. Pour hot TM into the vessels and allow it to solidify at room temperature. This process usually takes about 30 min.

*Note: Vessels can be stored at 4 °C, but due to the presence of organic nutrients, they are prone to contamination. Carefully inspect each vessel before use.*

**C. Preparation of the algal suspension and inoculation on BBM-coated slides**

**Figure 2.**
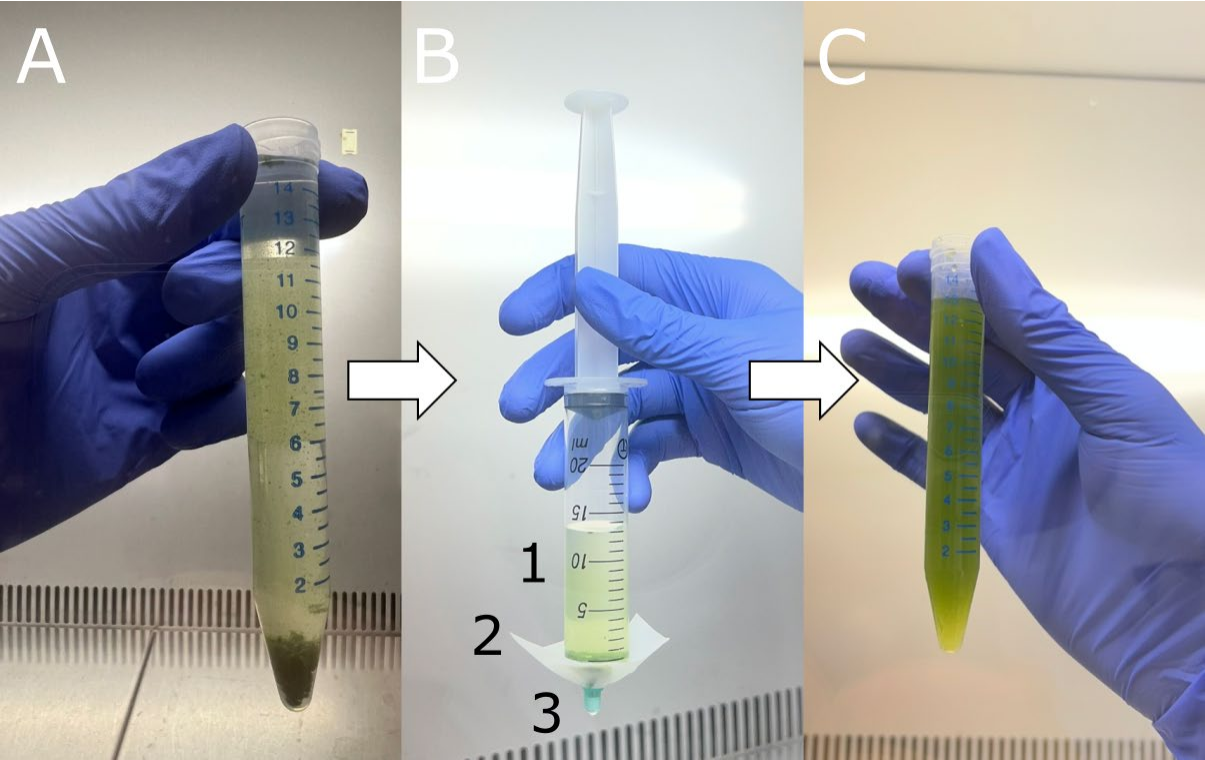
Homogenisation of the algal suspension. (A) Algal cells forming large clumps at the bottom of the Falcon^®^ tube containing liquid Bold’s basal medium (BBM). (B) Needleless syringe with the algal suspension ready to be homogenised. 1: algal suspension; 2: Nylon net of 40 μm mesh; 3: plastic plug of a syringe needle. (C) Homogenised algal suspension.

1. After 3 weeks of growth on solid TM under the conditions described in the Biological materials section, collect 0.4 g of cells from each *Trebouxia* vessel with a sterile disposable loop. The cell mass can be determined by transferring the collected material to a pre-weighed Falcon^®^ tube and measuring the fresh weight on an analytical balance.

2. Transfer 15 mL of liquid BBM (see Recipes) into a sterile 15 mL Falcon^®^ tube using a 0.5–5 mL pipette with autoclaved tips. To facilitate handling, first add 10 mL of BBM, then add the collected cells, and finally top up to the final volume of 15 mL with additional BBM. The algal cells will be clumped together. Depending on the species, the clumps will be smaller or larger (Figure 2A).

3. Attach a piece of nylon net of 40 μm mesh to the tip of a needleless syringe and secure it with the plastic plug of a fitting syringe needle (Figure 2B).

4. Remove the plunger from the syringe and pour the algal suspension into the syringe. Gently homogenise the suspension by pushing it through the nylon net with the syringe plunger (Figure 2C). Apply only light pressure to avoid damaging the cells.

*Note: This step breaks apart large aggregates of cells and ensures an even distribution of cells for inoculation. If the aggregates are too large, filter the suspension through a 100 μm nylon mesh first.*

5. Inoculate 5 μL of the algal suspension onto the surface of a BBM-coated slide, prepared as described in section A, using a 0.5–10 μL pipette with autoclaved tips. Each inoculum should be placed as a small droplet, which limits the area to be observed under the microscope. Two or three inocula can be placed on the same slide without risk of cross-contamination, as the BBM layer prevents liquid spreading.

**Critical:** Do not cover the inoculated area with a coverslip to avoid uncontrolled spread of flagellate cells.

6. Place the slides back into Petri dishes lined with moist adsorbent paper and incubate in a thermostatic chamber at 20 ± 1 °C, under 23 ± 1 μmol photons·m^-2^·s^-1^, with a 12/12 h light/dark regime.

**D. Light microscopy observations and monitoring of selected cells**

1. Place the BBM-coated slides (prepared as in section A and inoculated as in section C) on the stage of a light microscope.

2. Scan the surface of the slide and identify regions of interest that contain cells or groups of cells to be observed during the experiment.

3. Take note of the coordinates of each selected area using the microscope’s coordinate system.

*Note: This step allows the same cells to be re-localised for subsequent observations.*

4. Acquire images and/or videos of the selected cells using the camera attached to the microscope.

*Note: If the microscope is not in a sterile environment (e.g., under a biological hood), sterility will be lost after the first observation. The presence of moist absorbent paper in the Petri dishes may already compromise sterility. However, as stated in section A, the system should be free of contamination for up to 14 days.*

5. Place the slides back into the Petri dishes lined with moist absorbent paper and continue the incubation under the same conditions, as described in section C.

6. Repeat the observations daily to monitor the same cell groups over time.

**Critical:** As the algal cells will continue to grow, the field of view will become increasingly crowded. Daily observations are recommended to ensure reliable identification of the monitored cell groups.

## Data analysis

**Figure 3.**
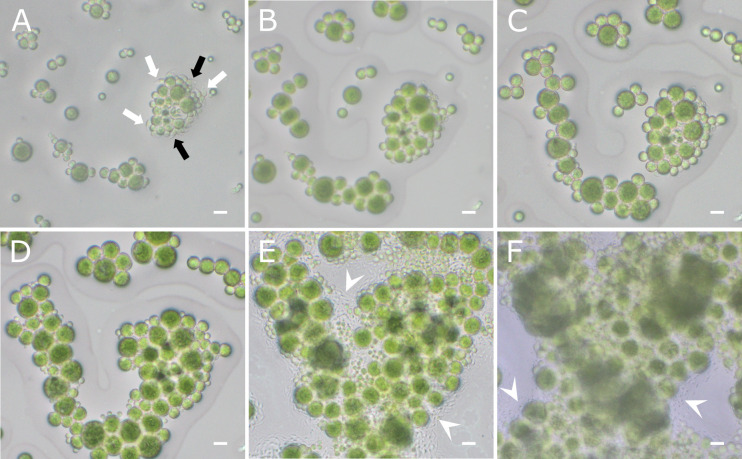
Representative images of *Trebouxia* flagellate cells over time. Flagellate cells of *T. angustilobata* observed under the light microscope at release (A) and after 3 (B), 4 (C), 8 (D), 15 (E), and 18 (F) days. White arrows indicate flagellate cells in clear focus and oriented with their major axis parallel to the field of view (suitable for analysis). Black arrows indicate flagellate cells that are partially visible or out of focus (to be excluded from analysis). White arrowheads indicate contaminations developing around the algal cells. Scale bars = 10 μm.

The most important outcome of this protocol is the ability to observe algal cells in real time over several days, allowing both qualitative observations (e.g., flagellate cells release, plasmogamic fusion, cell development) and quantitative analyses (e.g., cell size and morphology). In particular, the recording of videos at high frame rate, i.e., >25 frames per second, allows watching the video frame by frame, which increases the probability to analyse highly motile life stages in the correct positioning (e.g., when the major axis is parallel to the field of view) for morphometric characterisation (Figure 3).

Captured images and videos can be processed a posteriori with Fiji [12] or other software for automatic image analysis, for morphometric measurements (e.g., aspect ratio and circularity of flagellate cells). If statistical analysis of the cell populations is needed, data can be exported to R (v. 4.3.2; R Core Team, 2023) or comparable software for further analysis.

To obtain reliable results, at least 30–40 flagellate cells should be analysed for morphometric measurements and observation of cell development. Only cells with a clear focus should be included in the analysis (Figure 3A). Slides that are contaminated or dehydrated should be excluded from the analysis (Figure 3E, F).

The detailed morphometric and statistical analyses performed with this system and the observed cell development are described in [1] (see Materials and Methods “Morphological analysis of flagellate cells using light microscopy,” and “Statistical analyses”). Briefly, statistical analyses were performed in R software using generalised linear models (GLM) to test for infraspecific variability in the morphometric parameters of flagellate cells (i.e., aspect ratio and circularity). Gamma-distributed GLM were used for aspect ratio, and beta-distributed GLM were used for circularity. Model assumptions were checked through visual inspection of residuals, and dispersion models were applied when heteroscedasticity was detected.

## Validation of protocol

This protocol was used and validated in the following research article:

• Boccato et al. [1]. Zoospore diversity and sexual reproduction in the lichen-forming genus *Trebouxia*: From neglected motile cells to new evidence of sexual stages. *Plant Biology* (Figures 1–4, Figures S1–S2, Table S1, and Videos S1–S8).

The use of this system made it possible to successfully observe the development and behaviour of live flagellate cells over several days. The reproducibility of the method was demonstrated for four *Trebouxia* species (*T. decolorans, T. gelatinosa, T. vagua*, and *T. angustilobata*), belonging to three phylogenetic clades.

## General notes and troubleshooting

**General notes**

1. This protocol has been optimised for *Trebouxia* species, but, in principle, it can be applied to other unicellular and colonial green microalgae (e.g., Chlorophyceae, Trebouxiophyceae, and Ulvophyceae) when daily monitoring of specific life cycle stages is required. It is particularly suitable for studying the interactions and development of transient or motile stages (e.g., zoospores, gametes, and motile vegetative cells), which are often difficult to monitor in fixed samples, or for determining the developmental timing of cells.

2. A limitation of this system is that sterility cannot be fully ensured once the slides are observed outside a biological hood. Although this usually has no effect on short-term observations (up to 14 days), longer-term experiments could be affected by contaminating microorganisms. If another culture medium is used instead of BBM, especially a medium with organic nutrients, contamination could occur more quickly.
